# Measuring the Burden—Current and Future Research Trends: Results From the NIAAA Expert Panel on Alcohol and Chronic Disease Epidemiology

**DOI:** 10.35946/arcr.v35.2.16

**Published:** 2014

**Authors:** Rosalind A. Breslow, Kenneth J. Mukamal

**Affiliations:** **Rosalind A. Breslow, Ph.D., M.P.H., R.D.,***is an epidemiologist, Division of Epidemiology and Prevention Research, National Institute on Alcohol Abuse and Alcoholism, National Institutes of Health, Bethesda, Maryland.*; **Kenneth J. Mukamal, M.D.,***is associate professor of medicine, Harvard Medical School, Division of General Medicine & Primary Care, Beth Israel Deaconess Medical Center, Boston, Massachusetts.*

**Keywords:** NIAAA Expert Panel on Alcohol and Chronic Disease Epidemiology, alcohol consumption, alcohol burden, chronic disease, risk factors, epidemiology, research, diabetes, cardiovascular disease, cancer, stroke, liver disease, genetic factors, eating behaviors, clinical trials

## Abstract

Alcohol has a significant impact on health and well-being, from the beneficial aspects of moderate drinking to the detrimental effects of alcoholism. The broad implications of alcohol use on public health have been addressed through a wide range of epidemiological and clinical studies, many of which are described in this issue of *Alcohol Research: Current Reviews.* Where chronic disease is involved, alcohol use can be a risk factor that not only affects the onset of various chronic diseases but also exacerbates the ongoing extent and severity of those diseases. Lifestyle choices and genetic influences also contribute to, or help to alleviate, that risk.

Research is continuing to investigate how alcohol impacts chronic disease. The National Institute on Alcohol Abuse and Alcoholism (NIAAA) hosted a 2-day Expert Panel on Alcohol and Chronic Disease Epidemiology in August 2011 to review the state of the field on alcohol and chronic disease. The panel was chaired by Kenneth J. Mukamal, M.D., and Rosalind A. Breslow, Ph.D., M.P.H., R.D., and was convened by NIAAA’s Division of Epidemiology and Prevention Research.

Panel members (see textbox) represented a wide range of backgrounds and expertise, ranging from alcohol-related chronic diseases and risk factors to methods and technology. Among the chronic diseases addressed were diabetes, cardiovascular disease, cancer, stroke, and liver disease. The broader aspects of the design and implementation of clinical trials and the implication of technological advances for research also were considered. Other topics included the links between genetics and other lifestyle factors, such as eating behavior, and the relationship between drinking and various chronic diseases. Taken together, these summaries provide unique insight into the current state of research on alcohol’s role in chronic disease and the direction these investigations may take in the future. (For more information on the epidemiological challenges of elucidating the effects of alcohol consumption and drinking as they relate to the initiation/exacerbation and treatment of chronic diseases, see the article by Shield and colleagues [pp. 155–173]). Panel members also were asked what research they would most strongly support if funds were unlimited and how they might scale back that research if funding were limited (see Future Ideas textbox). Highlights from this panel are presented below and specific recommendations are listed in the accompanying sidebar.

## Clinical Trials

Clinical studies include clinical nutrition studies, controlled feeding studies, and metabolic studies. This type of research has numerous strengths for studying alcohol and chronic disease, including the ability to control alcohol dose and diet, collect abundant biologic samples from a variety of tissues, assess cause and effect, and examine mechanisms—all with a relatively small number of participants enrolled for a short period of time.

Clinical study end points typically are surrogate markers for chronic diseases because the disease itself may take years or even decades to develop. For example, lipoproteins and markers of inflammation have been used as surrogates for cardiovascular disease, insulin sensitivity for diabetes, and DNA damage for cancer.

According to Dr. David J. Baer, considerable need for controlled clinical studies on alcohol and chronic disease still exists. There have been few clinical studies, even on cardiovascular disease (Brien et al. 2011), which is the focus of most alcohol-related chronic disease research. He also noted the relatively few controlled clinical studies of alcohol and obesity (Sayon-Orea et al. 2011) that were advocated by the Report of the Dietary Guidelines Advisory Committee on the Dietary Guidelines for Americans (U.S. Department of Agriculture 2010).

Dr. Baer suggested the following future opportunities for alcohol and chronic disease research:

- Drinking patterns;
- Effects on metabolism and disease risk;
- Non-ethanol components of alcoholic beverages;
- Possible effects on cardiovascular disease, diabetes (insulin sensitivity), cancer, and bone metabolism;
- Gender and age differences (pre- and postmenopausal women, men);
- Genetic basis for response of chronic disease surrogate markers to alcohol;
- Energy metabolism, body weight regulation, and insulin sensitivity;
- Interaction of alcohol with lower-fat or higher-protein diets; and
- Bone metabolism.

## Cardiovascular Disease

Studies on alcohol and cardiovascular disease have yielded important findings with regard to public health. For example, we now know that the association of alcohol use within recommended limits with lower risk of heart disease depends more on the frequency with which alcohol is consumed and not on the type (Cleophas 1999). Wine, beer, and spirits all have been associated with reduced risk of myocardial infarction. Modest differences in the effects of those different types of alcohol are thought to be more a result of lifestyle differences among drinkers rather than a direct link to a specific type of alcohol. How often people drink alcohol has a larger impact on cardiovascular disease. Among men, drinking more frequently seems to have a greater impact than the actual amount consumed (Mukamal et al. 2003); effects are less clear among women. The beneficial effects of alcohol also have been shown to be similar for people with existing cardiovascular disease or diabetes (Costanzo et al. 2010; Koppes et al. 2006) and those in the general population. In addition to its beneficial effects on coronary heart disease, moderate drinking has been found to reduce the risk of ischemic stroke but at a lesser magnitude and with lower levels of consumption (Klatsky et al. 2001).

Although the exact mechanisms involved in these cardio-protective effects still are under investigation, the putative benefits on cardiovascular disease likely are the result of alcohol’s effects on lipids and insulin sensitivity (Dijousse et al. 2009).

In his presentation, Dr. Kenneth J. Mukamal noted that standard epidemiologic studies of alcohol consumption and coronary heart disease incidence or mortality are no longer useful, as virtually all prospective studies performed since 1980 have shown that moderate drinking reduces risk (Corrao et al. 2000; Mukamal et al. 2010; Ronksley et al. 2011). Recent analytic strategies have resulted in more precise statistical estimates, but the conclusion is unchanged. In essence, he stated, “We’ve been doing the same epidemiology since 1992.”

Dr. Mukamal suggested the following future opportunities for alcohol and cardiovascular disease research:

- Effects of heavy and binge drinking;
- Effects of changes in alcohol consumption over time;
- Differences in effect of gender-specific drinking patterns;
- Genetic interactions;
- Studies of new mechanisms directly related to alcohol’s effects (for example, cholesterol efflux capacity) (Khera et al. 2011);
- Pooling projects for questions that require large samples; and
- Use of case crossover designs to account for both triggering events and chronic use (Mostofsky 2011).

## Cancer

Alcohol consumption increases the risk for several cancers, including breast, colon, liver, and upper aero-digestive cancers (oral, pharynx, larynx, and esophagus) (Schutze et al. 2011; World Cancer Research Fund 2007). The potential mechanisms underlying alcohol’s effects include the carcinogenicity of acetaldehyde (for colorectal cancer and upper aero-digestive tract cancers), which is an intermediate product of alcohol metabolism; impairment of the one-carbon nutrient metabolism (for colorectal cancer); alteration of hormone levels (for breast cancer); and oxidative stress resulting from alcohol metabolism.

Dr. Edward Giovannucci noted the paucity of research on drinking patterns and cancer. He acknowledged too that studies can yield disparate findings, describing a study that initially showed no relationship between average alcohol consumption and prostate cancer but which in a posteriori analyses hinted at a possible relationship with high-quantity/low-frequency drinking (Platz et al. 2004).

In identifying areas for future research, Dr. Giovannucci discussed the importance of studying cancer–nutrient interactions, particularly for colon cancer. For example, the epidemiologic literature has consistently shown an interaction between alcohol and folate, a nutrient that seems to be protective at higher levels of drinking (Ferrari et al. 2007; Jiang et al. 2003). This suggests that the excess risk of cancer resulting from alcohol use potentially could be modified by a nutrient or combination of nutrients.

Further study also is needed to better understand the role of genetics and family history in cancer risk. The genes involved in alcohol metabolism (Yokoyama et al. 2001) and nutrient metabolism (for example, the gene *methylenetetrahydrofolate reductase* [*MTHFR*] for folate as well as other genes involved in the one-carbon metabolism pathway) are other areas that warrant additional study. Determining the molecular characteristics of tumors, such as tumor subtypes classified by level of methylation, which might reflect defects in one-carbon metabolism (Schernhammer et al. 2010), is another area that requires further investigation. In addition, little research has been conducted with cancer survivors, a group that may be especially willing to modify their drinking habits.

**Future Research Ideas, Large and Small, for Consideration**

In addition to the full panel discussions, panelists were asked to consider directions for future studies—both large and small. Specifically, the panelists described what studies they would suggest for future research and how they would refine those visions when funds are limited. Selected noteworthy examples are described below.

- A randomized trial to evaluate alcohol consumption and risk of multiple clinical outcomes with sufficient power to evaluate prespecified genetic environmental interactions would be ideal. However, with limited resources, it might be more realistic to use a hybrid design, with a prospective cohort study and a smaller nested trial. For example, a trial might evaluate if recommending moderate alcohol consumption, versus no recommendation, had an effect on cardiovascular and stroke outcomes among patients with a high risk for vascular problems.
- Clinical trials to establish the effects of alcohol consumption on clinical cardiovascular and cancer outcomes. A large-scale trial using high-risk populations with standardized exposure to alcohol would be ideal. A more practical approach would be to conduct shorter trials with subclinical measures of both cardiovascular disease and, to a lesser degree, cancer, using such techniques as serial computed tomography angiography and colonography.
- Studies to identify factors that influence the risk for liver disease among moderate drinkers. A large, prospective study would be ideal and would include serial measures of genomic, dietary, anthropometric, and behavioral risk factors obtained as objectively as possible, coupled with serial noninvasive measures of liver disease using magnetic resonance imaging for fat and fibroscan for fibrosis. Such a cohort could additionally fold in cardiovascular disease risk factors and clinical and subclinical cardiovascular disease. Among other things, this study would help to address the simultaneous associations of alcohol consumption with lower risk of cardiovascular disease but higher risk of fatty liver, which is associated with a higher risk for cardiovascular disease. Although of more limited utility, a cross-sectional study with the same measures would also be of clear import.
- Studies to verify estimates of drinking patterns. This is particularly important as self-reported estimates form the basis for epidemiological studies but have yet to be validated, particularly in the context of eating patterns, portion sizes, and health beliefs.
- Studies of how alcohol ingestion impacts energy balance in both moderate and binge drinkers.
- Studies to better understand the risk factors underlying alcohol-related chronic disease. These factors range from fixed characteristics, such as genetics and ethnic background, to broader modifiable behaviors, such as diet, exercise, or smoking. An ideal study would be multifaceted and include both disease-specific and composite global endpoints, such as healthy aging or survival free of chronic disease. A more limited study could simply compile data from the dozens of cohort studies worldwide where much of this data already have been collected. A more comprehensive effort would use ongoing studies prospectively to incorporate novel measures of drinking patterns, biomarkers of health status, or greater assessment of quality of life and mental health.

Finally, as noted by Dr. Giovannucci, alcohol increases the risk for many cancers, but not all. Recent studies have found that alcohol is associated with a lower risk of kidney cancer (Lee et al. 2007) and non-Hodgkins lymphoma (Kroll et al. 2012). Understanding how these two cancers differ from others is another area requiring additional research.

Dr. Giovannucci suggested the following future opportunities for alcohol and cancer research:

- Effects of drinking patterns on cancer risk;
- Nutrient interactions;
- Genetic susceptibility (genes related to alcohol metabolism, genes related to one-carbon metabolism);
- Tumor subtypes;
- Cancer survivors; and
- Pathways that might explain the limited protective aspects of alcohol consumption.

## Diabetes

Evidence that alcohol can impact diabetes has been consistent over several studies. Results from the Nurses’ Health Study (Stampfer et al. 1988), the Health Professionals Follow-up Study (Conigrave et al. 2001), a systematic review (Howard et al. 2004), and two meta-analyses (Baliunas et al. 2009; Koppes et al. 2005) all show that moderate drinking is associated with a lower risk of diabetes. Heavy drinking, on the other hand, seems to lead to an increased risk of diabetes, although sample sizes generally have been too small to draw firm conclusions.

Dr. Eric Rimm described specific areas of research that warrant further study. For example, only about 30 to 50 percent of alcohol’s beneficial effects on diabetes can be linked to biomarkers studied to date. In addition to its overall effect on insulin sensitivity (Davies et al. 2002), moderate alcohol consumption improves adiponectin, a fat-tissue hormone associated with insulin sensitivity; inflammatory status (Joosten et al. 2008); and HDL cholesterol. With regard to metabolic studies, he noted the value of using short-term feeding studies because they provide an opportunity to control and simultaneously examine drinking (for example, with meals or without) and diet (for example, high versus low glycemic load) (Mekary et al. 2011). He also discussed the importance of studying genetic predisposition (Beulens et al. 2007).

In addition to these areas, Dr. Rimm suggested several future opportunities for alcohol and type 2 diabetes research:

- Pool large cohort studies to maximize power to look at subpopulations where alcohol may be most detrimental or most beneficial.
- Pool data from large cohort studies with genetic information on alcohol metabolizing and diabetes-related genes to examine the interactions between alcohol, genetic predisposition, and diabetes risk.
- Conduct metabolic studies specifically within subgroups to examine how alcohol modifies risk based on lifestyle characteristics, such as body mass index, diet, and physical activity.

## Stroke and Cognition

Several important findings on the effects of alcohol consumption on the incidence of stroke have emerged from the Northern Manhattan Study, a prospective, multiethnic cohort study (Elkind et al. 2006; Sacco et al. 1999). In that study, subjects with the lowest risk for ischemic stroke consumed, on average, two drinks per day. Those effects were similar among drinkers of wine, beer, and liquor. In contrast, no protective effect was found for hemorrhagic stroke.

The study’s principal investigator, Dr. Ralph Sacco, presented the results of two meta-analyses. One found the greatest protection against all strokes combined was most evident at a lower level of drinking, less than or equal to one drink per day (Ronksley et al. 2011). Other analyses compared results from ischemic with hemorrhagic strokes (Reynolds et al. 2003). For ischemic stroke, moderate drinking was protective, whereas heavy drinking was associated with an increased risk; for hemorrhagic stroke, heavy drinking increased risk (although sample size was insufficient to study the effects of moderate drinking on hemorrhagic stroke).

The heterogeneity of strokes underscores the importance of studying stroke subtypes. Both ischemic strokes (the majority of all strokes) and hemorrhagic strokes (about 17 percent of all strokes) have subtypes with differing etiologies that may respond differently to alcohol consumption. Little research has been conducted on these subtypes, partly because of the small numbers of each that occur within most studies and the need for relatively large samples to obtain sufficiently precise estimates of risk. Numerous subclinical markers of stroke, such as endothelial function, currently are being pursued by researchers (Suzuki et al. 2009).

### Cognition

The prevalence of cognitive impairment is growing rapidly as the population ages, and, like stroke, cognitive impairment is not a single disease or condition. Studies of alcohol use and cognition have examined a variety of outcomes, including Alzheimer’s disease, cognitive function, dementia, and mild cognitive impairment (Lee et al. 2010). Studies and meta-analyses generally show that moderate drinking is associated with a decreased risk of dementia (Mukamal et al. 2003*b*; Peters et al. 2008), Alzheimer’s disease (Peters et al. 2008), vascular dementia (Peters et al. 2008), and cognitive decline (Peters et al. 2008). According to Dr. Sacco, there currently is great interest in vascular risk factors for dementia, yet little alcohol research has been done in that area.

**Recommendations for Strengthening Studies**

In addition to offering ideas for future studies, the Expert Panel also made recommendations for strengthening research in the field. Specific suggestions include:

1. Standardize alcohol consumption measurement in prospective and retrospective studies of alcohol and chronic disease to the greatest degree possible. Standardized measures:
  - Should include consumption quantity, frequency, and binge drinking (i.e., basic drinking patterns).
  - Should consider drinking over the lifespan (for example, during youth, middle age, menopause, and during time of heaviest drinking) as the critical time periods for effects of alcohol on chronic disease development are uncertain.
  - Are available from NIAAA and from the NIH/National Human Genome Research Institute Phenx Toolkit: http://www.niaaa.nih.gov/Resources/ResearchResources/Pages/TaskForce.aspx; https://www.phenx.org/Default.aspx?tabid=36
2. Strongly encourage collection of biological material and broad consent for genetic studies in all clinical trials and in as many population studies as possible.
3. Objectively validate standardized alcohol measures using novel technologies as they become available. Examples may include implantable biosensors and point-of-care devices with wireless transmission capability.
4. Develop new biomarkers for moderate alcohol consumption to complement those used for heavy drinking.
5. Identify surrogate markers for chronic disease (including measures of subclinical disease) that will have utility in small-scale studies and for elucidating mechanisms and pathways linking alcohol to chronic disease.
6. Pool data from existing cohort studies to facilitate examination by population subgroups, including but not limited to age, lifespan phase, race/ethnicity, menopausal status, body mass index/anthropometrics, dietary intake/nutritional status, smoking status, physical activity/fitness, cancer survivorship, and age of drinking onset. Pooled data also may facilitate studies of rare or understudied outcomes such as liver disease.
  - Standardized alcohol questions should be used where possible.
  - Confounding and interaction should be considered to ensure robust estimates and define susceptible subgroups.
  - Targeted sub-studies within large cohorts should be considered as a cost-efficient way to better understand and explain results in the full cohort. For example, when data on alcohol consumption are not gathered in enough detail in the original study, targeted follow-up studies may be used among stratified subsets of subjects to collect biological samples and to obtain more detailed data on consumption for extrapolating to the parent study.
7. Include associations between alcohol dependence/abuse and chronic disease outcomes. Studies using pooled data or sub-studies within large cohorts may have the power to address these drinking problems. Data on period of maximum drinking could be important, particularly given the marked variation in alcohol intake during the lifespan.
8. Perform studies in understudied areas, including but not limited to the effects of alcohol on diabetes, obesity, cognition, healthy aging, and food intake.
9. Focus on relationships between drinking patterns and chronic disease. Drinking patterns include but are not limited to basic patterns such as usual quantity, frequency, and binge drinking as well as when, where, and with whom alcohol was consumed and whether it was consumed with a meal.
10. Encourage clinical trials across the spectrum of chronic disease from studies that examine key physiological parameters and intermediate studies such as feeding studies that examine surrogates or subclinical phenotypes to practical trials that examine chronic disease outcomes.
  - Physiologic studies are preferred when epidemio-logic evidence is relatively limited.
  - Practical trials are preferred when there is extensive evidence from physiological and epidemiological studies.
11. Encourage studies examining the interactions between the genetics that predispose individuals to drink and the genetics that modify how alcohol affects chronic disease.
12. Encourage studies of carefully defined homogeneous phenotypes. For example, studies are needed to clarify the effects of alcohol on thrombotic versus embolic ischemic stroke, Alzheimer’s disease versus other dementias, specific eye diseases, etc.
13. Encourage studies on moderate drinking patterns and metabolism ranging from total energy and macronutrient metabolism to specific metabolic pathways for small molecules such as vitamins, amino acids, sugars, and steroids and their products and precursors.
14. Examine the effectiveness of communication messages about drinking. Studies may include, but are not limited to, how to disseminate cost-benefit messages, individualized messages based on patient demographic and clinical history, and guidance for health care professionals on how to advise patients.
15. Encourage the use of natural experiments to examine whether policy interventions or alcohol intervention studies might change the relationship between alcohol and chronic disease.

Other future opportunities for research into alcohol and chronic neurological disease noted by Dr. Sacco include the following:

- Cohort studies with careful end point adjudication to separate ischemic stroke subtypes and different etiologies of dementia and cognitive impairment;
- Examination of interactions with race and ethnicity and other neurological risk factors;
- Comparison of associations across beverage types for neurological outcomes; and
- Understanding protective alcohol mechanisms including inflammatory relationships, subclinical measures and biomarkers, and gene–environment interactions.

## Chronic Liver Disease

Chronic liver disease has long been associated with alcohol consumption and includes alcoholic liver disease, hepatitis C, and nonalcoholic steatohepatitis. Despite this clear association, however, there is a lack of strong clinical measures to describe and predict the progression of chronic liver disease. Dr. James Everhart noted that the course of alcoholic liver disease is several decades in duration and begins as simple steatosis (fatty liver) before progressing to more advanced stages including steatohepatitis, alcoholic cirrhosis, and, eventually, liver failure.

Dr. Everhart noted that alcoholic liver disease may be overrepresented in terms of mortality because of the current classification system. Histologically, alcoholic fatty liver and nonalcoholic fatty liver look similar (Scaglioni et al. 2011), and patients with otherwise similar multiple risk factors and histology may be classified as having alcoholic liver disease rather than nonalcoholic steatohepatitis simply because they do or do not drink. According to Dr. Everhart, the current strict separation of alcoholic and nonalcoholic fatty liver disease limits epidemiology, public health, and clinical understanding.

In examining the effects of drinking amounts on liver disease, little association has been found between moderate drinking and alcoholic liver disease, and only a minority of very heavy drinkers develops alcoholic liver disease, although the reason is not clear. It is possible that drinking patterns and diet each play a role in risk. More information also is needed to determine if drinking at times other than during meals could increase risk.

Other factors that put people at higher risk for liver disease include being obese, using cannabis, having diabetes, and being female (Hart et al. 2010). Conversely, coffee consumption seems to lower risk and smoking seems to have no effect on the development of chronic liver disease. Genetic susceptibility is another important risk factor for liver disease. For example, a variant in one gene, *PNPLA3*, originally associated with fatty liver, has been strongly associated with alcoholic liver disease. Again, additional research is needed to determine how these factors influence alcohol’s effects.

Dr. Everhart suggested several future opportunities for alcohol and chronic liver disease research:

- Improve the current chronic liver disease classification scheme;
- Develop reliable and accurate measures of progressive liver disease that can be applied serially;
- Implement better measures of alcohol consumption and its patterns to study drinking patterns and interactions between drinking and diet;
- Evaluate how genetics may influence the link between alcohol consumption and the risk of liver disease; and
- Identify determinants of chronic liver disease among heavy drinkers.

## Genetics

Chronic diseases tend to run in families yet do not follow a simple genetic pattern; that is, they are complex and polygenic. Identifying the genes that affect chronic disease risk can be hampered by multiple factors, including phenotypic complexity, multiple genes with small effects, environmental variability, gene–gene interactions, and gene–environment interactions. Alcohol’s role in chronic disease likely reflects a gene–environment interaction in which risk is influenced by genes, by lifestyle choices, and by a combination of both. In addition, as noted by Dr. Howard J. Edenberg, most of the variations in genes related to alcohol and chronic disease likely have only small effects, making those genetic influences especially difficult to identify.

One way of overcoming these difficulties, as proposed by Dr. Edenberg, is to obtain large sample sizes by combining data from multiple epidemiologic studies. This enables investigators to examine gene–environmental associations using secondary data analyses. The drawback is that studies typically ask different questions about alcohol use and often include different time frames, often collect no data on drinking problems, and may not obtain appropriate consent for genetic testing. Dr. Edenberg suggested a number of strategies to manage these obstacles. For example, investigators could be encouraged to incorporate standardized alcohol consumption questions, particularly for patterns of consumption, and to obtain DNA samples using proper consent for genetic studies, where appropriate. Existing studies also could be enhanced through targeted ancillary studies in which key subsets of subjects are re-contacted to provide more detailed or standardized information. The payoffs from such steps could lead to the discovery of key genes and pathways that reveal mechanisms and potential targets for therapy. Even if the effect of a variant is small, the pathway it leads to could be of major importance.

Dr. Edenberg suggested several future opportunities for the genetics of alcohol and chronic disease research:

- Design and incorporate more detailed alcohol exposure measures that include patterns of consumption and drinking problems;
- Search out ongoing and planned studies to;
  – Partner to incorporate exposure measures as early as possible;
  – Target follow-up and additional studies to gather more detailed exposure information and genetic samples; and
  – Encourage collection of samples with consent for genetic studies.

## Eating Behaviors

The link between alcohol intake and eating behaviors is not well known. Studies generally show that alcohol calories, when added to the diet, increase total energy intake (Yeomans 2010). Yet despite the fact that alcohol is an energy source, is largely uncompensated (i.e., supplements rather than replaces other calories), may weaken feeding controls, and spares fat for storage, little evidence exists that moderate drinking is associated with increased body mass index or weight gain (Liangpunsakul 2010; Liu et al. 1994; Wang et al. 2010) (although a French study did show such an effect [Lukasiewicz et al. 2005]). On the other hand, certain drinking patterns, particularly binge drinking, have been associated with higher body mass index (Arif and Rohrer 2005; Breslow and Smothers 2005), although impulsivity related to both eating and drinking could be an alternative explanation. According to Dr. Richard Mattes, determining alcohol’s effects on eating behaviors is further confounded by beverage consumption itself and the fact that energy compensation for fluids is less than for semisolid or solid foods (Mattes 1996; Mourao et al. 2007).

He also suggested that what people think they are eating may be more important in terms of appetitive sensations than its true energy value, noting current research showing that manipulating food form (liquid or solid) can alter a person’s expectation of how filling that food will be.

Dr. Mattes suggested several research opportunities for future studies on ingestive behavior and alcohol-related chronic disease research, particularly in controlled experimental designs:

- Clarify the role of moderate alcohol consumption on energy balance;
- Assess which properties of alcohol contribute to hunger and satiety;
- Ascertain the true biological energy value of alcohol;
- Test the role of drinking patterns on energy balance; and
- Determine the effects of different levels of alcohol consumption on body composition and energy balance.

## Technology

A number of promising technologies and medical devices currently are under development by the National Institute of Biomedical Imaging and Bioengineering and others that may enhance alcohol-related chronic disease research in the future. Dr. John Haller reviewed the research on three areas: sensors, point-of-care (POC) diagnostic devices, and imaging technologies and bioinformatics tools.

Sensors are used to detect and quantitate clinically relevant analytes. Examples include BioMEMs, microfluidics (Chin et al. 2011), and nanoscale technologies, including micro-total analysis systems, arrays, and biochips. These multifunctional devices can measure multiple analytes across a variety of diseases using a platform the size of a credit card.

Such technologies then can be combined into POC tests, which are defined as diagnostic testing at or near the site of patient care (rather than at centralized laboratories). Benefits include earlier diagnosis of disease and the ability to monitor patients at home. For example, POC tests for alcohol include a breath test and saliva-testing devices (http://www.aacc.org/events/online_progs/documents/AlcoholTesting1.2.pdf); SpectRx, a wristwatch-type device; and Giner, a WrisTas transdermal sensor for measuring alcohol consumption (Marques and McKnight 2009). Dr. Haller also reviewed implantable monitors and a tattoo using nanosensors that reside under the skin. By shining a light on the tattoo the subject enables tracking of sodium and glucose levels by portable digital devices, including smartphones. In the future, such a technology could be used to track alcohol consumption.

Biomedical imaging of the brain is another area where advances could be applied to the study of alcohol and chronic disease. Most radiology images (e.g., magnetic resonance imaging [MRI], computerized tomography) show anatomy/morphology. These images generally capture the late stages of chronic disease. An alternative approach would be to examine the physiological function (e.g., neuroreceptors) using nuclear imaging (e.g., positron emission tomography and single-photon emission computed tomography). Magnetic resonance spectroscopy can image relative chemical composition. MRI diffusion tensor imaging can image white matter tracts (connectivity), and functional MRI can image relative blood flow, a marker of neural activity. These structural and functional neuroimaging methods currently are being used in alcohol research (Buhler and Mann 2011). Dr. Haller noted that informatics (data modeling, simulation, and analysis) also will have a significant role in making sense of the large amounts of high-dimension data now available.

Dr. Haller had the following suggestions regarding alcohol-related chronic disease research:

- Among the variety of technologies and medical devices that exist for the study of individuals and populations, those of particular interest might include sensors, POC diagnostic devices, imaging technologies, and bioinformatics tools;
- A better alternative to the “hammer-in-search-of-a-nail” approach in imaging is to define the clinical problem of interest first, then find the appropriate tools to address the problem or chronic disease under study;
- Alcohol and chronic disease epidemiology could be improved through the use of new sensors (including POC diagnostics, sensors embedded in the home or implanted in the body) to enhance alcohol measurement and by techniques that can image physiological function early in the course of chronic disease; and
- Technological advances will inevitably produce vast amounts of data about individuals and populations, but they require new informatics tools that enable meaningful use of the data in wide varieties of research settings.

## Summary

This NIAAA workshop provided an excellent forum for summarizing the current state of the field and for identifying future research opportunities. Although by no means exhaustive, the ideas provided here highlight areas in need of additional study and offer a roadmap for moving forward across a variety of methodological approaches and content areas. NIAAA would like to thank all of the presenters for their insight and for taking the time to participate in this unique workshop. Our hope is that the ideas presented here will stimulate additional research and further advance our understanding of the role of alcohol in chronic disease.

## Additional Resources

The agenda, roster of speakers, and speaker’s abstracts can be obtained from the author. A copy of the meeting transcript also is available from the author, upon request.
